# Effects of feeding injury from *Popillia japonica* (Coleoptera: Scarabaeidae) on soybean spectral reflectance and yield

**DOI:** 10.3389/finsc.2022.1006092

**Published:** 2022-10-20

**Authors:** Arthur V. Ribeiro, Theresa M. Cira, Ian V. MacRae, Robert L. Koch

**Affiliations:** ^1^ Department of Entomology, University of Minnesota, Saint Paul, MN, United States; ^2^ Department of Entomology, University of Minnesota, Northwest Research and Outreach Center, Crookston, MN, United States

**Keywords:** herbivory, integrated pest management, Japanese beetle, remote sensing, yield

## Abstract

Remote sensing has been shown to be a promising technology for the detection and monitoring of plant stresses including insect feeding. *Popillia japonica* Newman, is an invasive insect species in the United States, and a pest of concern to soybean, *Glycine max* (L.) Merr., in the upper Midwest. To investigate the effects of *P. japonica* feeding injury (i.e., defoliation) on soybean canopy spectral reflectance and yield, field trials with plots of caged soybean plants were established during the summers of 2020 and 2021. In each year, field-collected *P. japonica* adults were released into some of the caged plots, creating a gradient of infestation levels and resulting injury. Estimates of injury caused by *P. japonica*, ground-based hyperspectral readings, total yield, and yield components were obtained from the caged plots. Injury was greatest in the upper canopy of soybean in plots infested with *P. japonica*. Overall mean canopy injury (i.e., across lower, middle, and upper canopy) ranged from 0.23 to 6.26%, which is representative of injury levels observed in soybean fields in the Midwest United States. Feeding injury from *P. japonica* tended to reduce measures of soybean canopy reflectance in near infra-red wavelengths (~700 to 1000 nm). These results indicate that remote sensing has potential for detection of injury from *P. japonica* and could facilitate scouting for this pest. Effects of *P. japonica* injury on total yield were not observed, but a reduction in seed size was detected in one of the two years. The threat to soybean yield posed by *P. japonica* alone appears minimal, but this pest adds to the guild of other defoliating insects in soybean whose combined effects could threaten yield. The results of this research will guide refinement of management recommendations for this pest in soybean and hold relevance for other cropping systems.

## Introduction

Japanese beetle, *Popillia japonica* Newman (Coleoptera: Scarabaeidae), is native to Japan and known to feed on more than 300 plant species (1). *Popillia japonica* has expanded its geographic range and became an invasive species in the United States in the early 1900s and Canada in 1998 (1, 2). More recently, *P. japonica* was also reported in mainland Europe (3) and it is now present in at least three European countries (4). Additionally, *P. japonica* has the potential to expand its range even further and invade Central and South America, Africa, and Oceania (5). In its region of origin, *P. japonica* is a minor agricultural pest, probably due to unfavorable environmental conditions and the presence of natural enemies (1). However, in invaded regions, *P. japonica* is an economically important pest of ornamental plants and turf in landscapes, and horticultural and field crops (1, 2, 6).

Soybean, *Glycine max* (L.) Merrill (Fabales: Fabaceae), is one of the most valuable crops worldwide due to its seed composition (i.e., oil and protein content) and versatility of end-use (7). The United States is the second largest soybean producer worldwide (8), but its production is compromised by the attack of insect pests (7). Defoliation caused by insect feeding is a common injury seen in soybean fields that can potentially lead to yield losses due to the reduction of plant photosynthetic area and disturbance of physiological processes (9). In soybean, *P. japonica* adults feed on leaves, creating a characteristic lace-like pattern of defoliation (6). Infestations of *P. japonica* alone can cause up to 20% loss of soybean yield (10), but *P. japonica* damage to soybeans can be even more problematic when combined with other defoliating insects (11). However, impacts of *P. japonica* feeding have not been well quantified in contemporary soybean varieties.

Current management of defoliating insects in soybean generally relies on the presence of the pest and estimation of percent defoliation across the whole field based on visual assessment of leaves from the top, middle, and bottom of plants selected from throughout the field (6). For *P. japonica*, assessment of the entire canopy of the crop is of particular importance because adults aggregate on the upper leaves of the plants and abundance tends to be higher at the edge of the fields, which can lead to overestimation of defoliation, especially at the field edges (6). Overall, traditional scouting and decision making for defoliating pests, like *P. japonica*, in soybean can be time consuming and therefore increase the overall cost of management.

Remote sensing became prominent in the past decades as a promising method for the detection and monitoring of plant stresses (e.g., insect feeding) (12–15). Remote sensing may be preferable to conventional scouting methods because it is faster and offers better coverage of the field (13, 14). Furthermore, remote sensing allows for early detection of diseases and pests (13). Typical applications of remote sensing consist of using sensors for contactless measurement of the electromagnetic radiation reflected from plants (12, 13). Numerous studies have documented the effects of pest injury and diseases on spectral reflectance of crops, mainly in the visible and near infra-red ranges of the electromagnetic spectrum (16–21). Studies assessing the effects of defoliation on plant spectral reflectance have focused mainly on forest areas (13–15), but field crops have also been investigated (22–26). In soybean, the normalized difference vegetation index (NDVI) was proposed for the detection of defoliation with simulated (22) and actual insect feeding by lepidopteran pests (25). More recently, Iost Filho et al. (2022) (26) evaluated the effects of defoliation by two lepidopteran pests on soybean leaf reflectance using individual wavelengths from the visible and near infra-red spectrum.

There is a lack of information of the effects of defoliation on the spectral reflectance of soybean that include individual wavelengths from the visible and near-infrared spectrum for other defoliators, such as *P. japonica*, especially under field conditions. Additionally, as abovementioned, a better understanding of the impacts of *P. japonica* feeding on yield of contemporary soybean varieties is also required. Thus, this study was done to assess the effects of feeding injury from *P. japonica* on the spectral reflectance, total yield, and yield components of soybean. Results of this study will help advance integrated pest management programs for *P. japonica* in soybean fields.

## Methods

### Field sites

This study was done in soybean fields of approximately 1 ha located at the University of Minnesota (UMN) Saint Paul campus (44.9898369° N, 93.1802096° W), and at the UMN Research and Outreach Center (44.7113597° N, 93.1041755° W) in Rosemount, Minnesota, United States, during 2020 and 2021, respectively. The soybean variety Stine ‘19EA32’ was planted on 15 May 2020 and the variety Golden Harvest ‘1012E3’ on 15 June 2021 with a seeding rate of 370,000 seeds/ha and row spacing of 0.76 m. When plants were at the V3 growth stage (plants with three fully expanded trifoliate leaves (27), plots of soybean were caged for manipulation of insect populations. Individual plots comprised two rows of soybean that were 1.5 m long (approximately 80 plants per plot), and caged with a 1.5×1.5×1.5-m polyvinyl chloride (PVC) frame covered with white no-see-um mesh (Quest Outfitters, Sarasota, FL, USA). A total of 32 and 24 plots (i.e., cages), arranged in 8 and 6 blocks, were caged in 2020 and 2021, respectively. Before caging, plants were visually inspected for the presence of insects and any individuals found on the plants were manually removed.

In each year, half of the plots in each block were randomly selected for infestation with field-collected *P. japonica* adults on four dates to create a gradient of insect injury. Adult *P. japonica* were collected from soybean fields using dual-lures (female sex pheromone and floral attractant) attached to Trécé Pherocon^®^ standard traps (yellow top and green vented catch can) (Trécé Inc., Adair, OK, USA). Two days prior to each infestation, *P. japonica* were collected from traps 4-5 times per day to reduce insect mortality due to excess heat inside the traps. Trapped *P. japonica* adults were transferred to 34.29×34.29×60.96-cm pop-up insect cages (Bioquip, Rancho Dominguez, CA, USA). As a food source, each cage contained 3-4 field-collected soybean stems cut at the soil level with the cut end of the stems placed inside 20-mL tubes with pierceable caps containing water. For infestations, live (i.e., actively moving) *P. japonica* adults were manually collected from the pop-up cages and placed in containers to be transported to the field. The number of *P. japonica* adults in each container was estimated based on fresh biomass using the methods of Ebbenga et al. (2022) (28) and an analytical scale (Sartorius ENTRIS224-1S, Sartorius Lab Instruments GmbH & Co. KG, Goettingen, Germany). In 2020, *P. japonica* were released on 28 July (1037 individuals per plot), 3 August (224 individuals per plot), 13 August (212 individuals per plot), and 24 August (39 individuals per plot) for a total of 1512 individuals per plot. Similarly, in 2021, *P. japonica* were released on 4 August (831 individuals per plot), 9 August (877 individuals per plot), 16 August (945 individuals per plot), and 24 August (80 individuals per plot) for a total of 2734 individuals per plot.

### Data collection

Spectral measurements were recorded within 2 h of solar noon (to reduce atmospheric and solar angle effects), with clear sky conditions or with low cloud cover (< 20%) and a clear view between the sun and the field. Measurements of canopy spectral reflectance were taken on 30 July and 4 September of 2020, and 17 August and 30 August of 2021, using a hyperspectral spectroradiometer (FieldSpec^®^ HandHeld 2™ VNIR spectroradiometer, ASD Inc., Boulder, CO, USA) able to detect wavelengths ranging from 325 to 1075 nm with accuracy of ±1 nm. On each sample date, the spectroradiometer was calibrated immediately before the beginning of measurements and every 7-10 minutes throughout data collection with a Spectralon^®^ reference standard (Labsphere, Inc. Sutton, NH, USA). In each plot, four spectral measurements were manually taken, two from each row, at approximately 0.5 m above the canopy. Cages were opened immediately before and closed immediately after measurements. Canopy-level spectral reflectance data were processed using the software ViewSpec Pro version 6.2.0 (ASD Inc., Boulder, CO, USA) and individual measures were averaged for each plot. Four vegetation indices were calculated from canopy-level spectral reflectance (**Table 1**) for each plot. These indices were selected because they were used in previous studies investigating the effects of insect feeding and diseases on soybean spectral reflectance (20, 29, 30).

**Table 1:** Vegetation indices tested in this study for the detection of effects of *Popillia japonica* feeding injury in plots of caged soybean plants in the field during 2020 and 2021 in Saint Paul, MN and Rosemount, MN, respectively.

Index	Name	Equation*	Reference
NDVI	Normalized Difference Vegetation Index	(R_800_-R_680_)/(R_800_+R_680_)	(20)
NDRE	Normalized Difference Red Edge	(R_750_-R_705_)/(R_750_+R_705_)	(29)
GNDVI	Green Normalized Difference Vegetation Index	(R_801_-R_550_)/(R_801_+R_550_)	(29)
MCARI	Modified Chlorophyll Absorption Reflectance Index	[(R_700_-R_670_)-0.2×(R_700_-R_550_)]×(R_700_/R_670_)	(30)

*R_x_, reflectance at wavelength x.

To avoid effects on canopy reflectance measures caused by disturbing the soybean plant canopies, inspection of plants for other insects was performed one day before or one day after collection of spectral data. To do so, whole-plant counts of other insects were recorded for five plants per plot, which were later averaged for each plot. Insects observed on the plants were not removed. Estimates of injury from *P. japonica* were done one day before or after measurements of canopy spectral reflectance. Five leaflets were randomly selected and collected from the lower, middle, and upper portions of the canopy of each plot (i.e., 15 leaflets/plot). These leaflets were placed in individually-labeled 17×17-cm resealable plastic bags, which were placed in a cooler with ice packs for transportation to the laboratory where they were stored in a refrigerator at 5°C to avoid desiccation. To quantify *P. japonica* feeding injury (i.e., percentage of leaflet area removed), leaflets were placed individually on a white surface and were fully extended and flattened under a transparent glass circle (180 mm diameter). Measurements of injury were performed on pictures of each individual leaflet using the software LeafByte version 1.3.0 (31) with an iPad (A1893, Apple Inc., Cupertino, CA, USA). Mean canopy injury (%) for lower, middle, and upper canopy and total canopy (i.e., across lower, middle, and upper canopy) were obtained for each plot.

On 9 October 2020, plants were hand-harvested and seeds obtained with a threshing machine (LPR UMB, Almaco, Nevada, IA, USA). On 19 October 2021, plots were harvested with a small plot combine. Seeds were then manually inspected to remove debris, placed in individual paper bags for each plot and brought back to the laboratory for assessment of yield. Total yield (ton per ha) was obtained by weighing all the seeds from a plot on a scale (Scout Pro SP 4001, Ohaus Corp., Pine Brook, NJ, USA). Seed size (g) was estimated by separately weighing three sub-samples of 100 seeds from each plot (i.e., 100-seed weight). Total number of seeds for each plot (i.e., seed number) was calculated using the total weight of seeds for each plot and the 100-seed weight.

### Data analyses

As mentioned, plants in the caged soybean plots were inspected for the presence of other insects one day before or one day after collection of spectral data. *Aphis glycines* Matsumura (Hemiptera: Aphididae) was present in some of the cages, but no honeydew was observed on the plants. For the purposes of the present study, plots with average *A. glycines* densities above 20 aphids/plant were removed to avoid potential confounding effects of *A. glycines* feeding on soybean spectral reflectance. This threshold was used because previous research indicated that densities lower than 20 aphids/plant have negligible effects on soybean canopy reflectance (20, 32). A total of 3, 8 and 7 plots with average *A. glycines* densities above 20 aphids/plant were removed from 4 September 2020, 17 August 2021 and 30 August 2021, respectively. Thus, the total number of plots analyzed was 16 uninfested and 16 infested with *P. japonica* adults on 30 July 2020, 15 uninfested and 14 infested on 04 September 2020, 7 uninfested and 9 infested on 17 August 2021, and 10 uninfested and 7 infested on 30 August 2021. However, to account for the potential effects of *A. glycines*, even at low numbers (i.e., densities lower than 20 aphids/plant), on the spectral reflectance of soybean, *A. glycines* density was also included in the analyses (see below).

All analyses were performed and graphs made using the software R version 3.5.1 (33) and RStudio Desktop version 1.1.463 (34). For each date in each year, stratum-specific injury (i.e., lower, middle, and upper canopy), total canopy injury, and spectral reflectance of the canopy were evaluated using variable dispersion beta regression models with a logit link function (package, *code*: betareg, *betareg* (35)). These response variables were included in the models as proportions (i.e., values between 0 and 1). For stratum-specific injury, infestation status (i.e., uninfested or infested with *P. japonica*), canopy stratum (i.e., lower, middle, or upper canopy) and their interaction were included as explanatory variables; and infestation status was included as an additional regressor for the estimation of the model precision parameter. For total injury, infestation status was used both as the explanatory variable and for the estimation of the model precision parameter. For spectral reflectance of the canopy, wavelengths and vegetation indices were analyzed separately, with total canopy injury used both as the explanatory variable and for the estimation of the model precision parameter. The inclusion of a precision parameter significantly improved the models, which was checked *via* a likelihood-ratio test comparing the full model with and without this parameter (lmtest, *lrtest* (36)). Block, *Aphis glycines* density and its interaction with total canopy injury were initially included in the models as explanatory variables, but they were overall non-significant (P > 0.05) and therefore removed from the models. Model assumptions were assessed with diagnostic plots of residuals. Similarly to Geissinger et al. (2022) (37), the significance of explanatory variables for stratum-specific injury was obtained *via* sequential nested likelihood-ratio tests (lmtest, *lrtest* (36)), and mean separation tests with *P*-values of pairwise comparisons adjusted with the Tukey method were done using estimated marginal means (α = 0.05) (emmeans, *emmeans* (38)). The significance of explanatory variables for total canopy injury and spectral reflectance of the canopy was estimated with partial Wald tests (stats, *summary* (33)).

Seed number, 100-seed weight, and total yield were analyzed with general linear models (stats, *lm* (33)) with injury (%) as the explanatory variable. Block was initially included in the models, but it was overall non-significant and therefore removed from the models. Linear model assumptions were visually checked with residual and quantile-quantile scatterplots, and formally with a global validation test (gvlma, *gvlma* (39)). The presence of outliers was assessed *a priori* with a Bonferroni outlier test (car, *outlierTest* (40)). One observation was indicated as an outlier and model assumptions were accepted after its removal.

## Results

### Feeding injury

**Figure 1:**
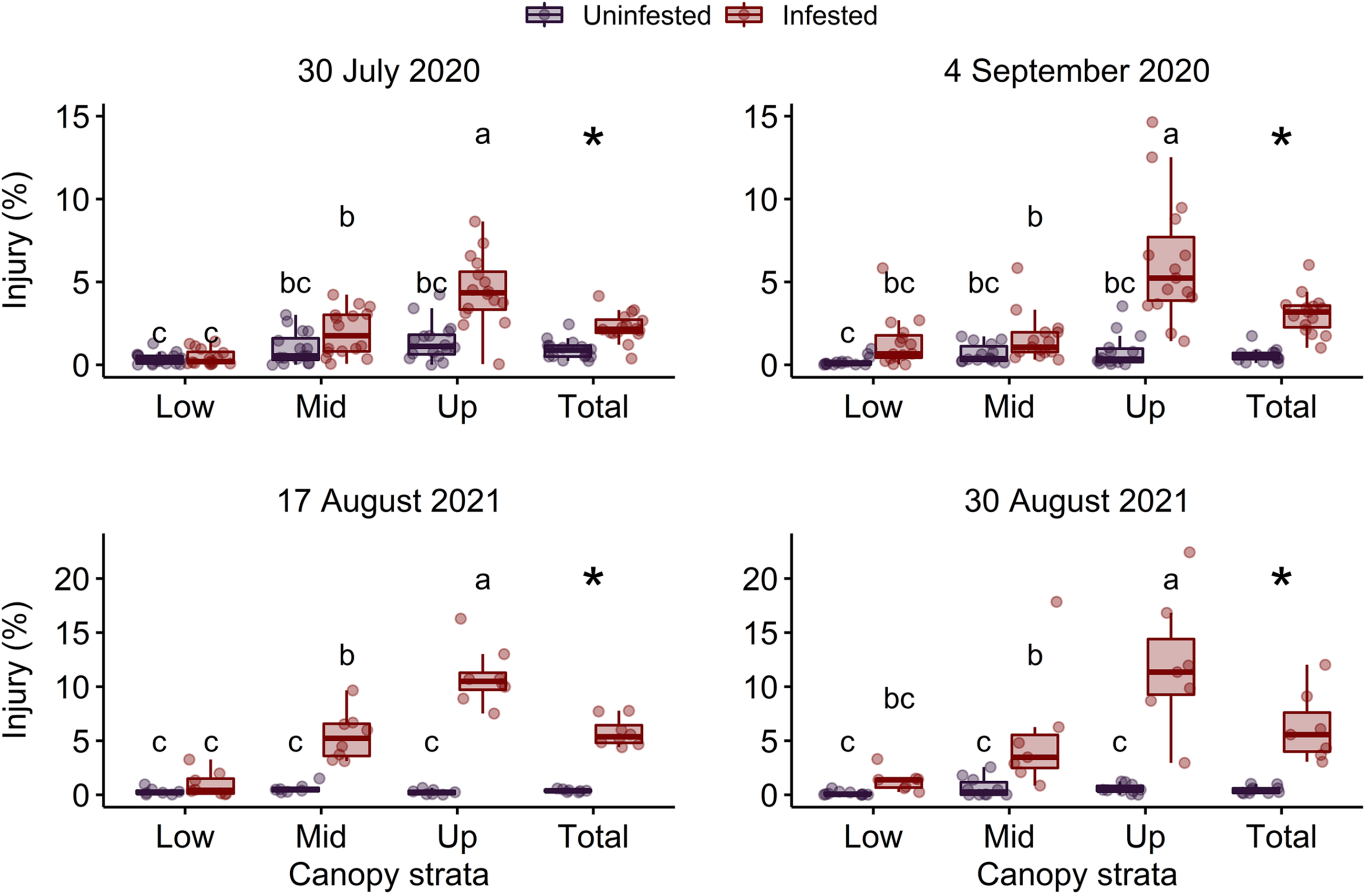
Percentage of leaf area injured in the lower (Low), middle (Mid), upper (Up) and total (i.e., across lower, middle, and upper) canopy in plots of caged soybean plants in the field that were uninfested or infested with *Popillia japonica* on two sample dates in each of 2020 and 2021. Different letters within each graph indicate significant differences among lower, middle, and upper canopy strata according to the Tukey’s test (P < 0.05). Asterisks indicate significant differences within each graph between infestation treatments for total canopy injury according to the Tukey’s test (P < 0.05).

The interaction between *P. japonica* infestation status and canopy stratum was significant on 4 September 2020 and 17 August 2021 (P < 0.001) (**Table 2**). However, on 30 July 2020 and 30 August 2021, the interaction between *P. japonica* infestation status and canopy stratum was not significant. On these two dates, canopy injury of soybean was significantly affected by *P. japonica* infestation status (P < 0.001) and canopy stratum (i.e., lower, middle, and upper canopy) (P < 0.001) (**Table 2**). Overall, injury in uninfested plots did not differ among canopy strata, but injury in plots infested with *P. japonica* was greater in the upper stratum of the soybean canopy (**Figure 1**). Across the two years, mean canopy injury ranged from 0.13 to 0.37%, 0.59 to 0.97%, and 0.25 to 1.40% in the lower, middle, and upper strata, respectively, in uninfested plots (**Figure 1**). For plots infested with *P. japonica* adults, mean canopy injury ranged from 0.95 to 1.35%, 1.58 to 5.45%, and 4.46 to 12.00% in the lower, middle, and upper strata, respectively, across the two years (**Figure 1**). Similarly, mean total canopy injury (i.e., across lower, middle, and upper strata of the canopy) ranged from 0.42 to 0.91% and from 2.28 to 6.51% in uninfested and infested plots, respectively, and was significantly higher in plots infested with *P. japonica* adults (P < 0.001) (**Table 2** and **Figure 1**).

**Table 2:** Likelihood ratio tests of beta regression models testing the effect of *Popillia japonica* infestation status (i.e., infested or unifested), canopy stratum (i.e., lower, middle, or upper canopy) and their interaction on feeding injury within the canopy, and of *P. japonica* infestation status on total feeding injury in plots of caged soybean plants in the field during 2020 and 2021 in Saint Paul, MN and Rosemount, MN, respectively.

Date	Within canopy			Total
	Infestation status	Canopy stratum	Interaction	Infestation status
30 July 2020	*χ* ^2^_(1)_ = 13.04 P < **0.001**	*χ* ^2^_(2)_ = 29.84 P < **0.001**	*χ* ^2^_(2)_ = 4.77 P = 0.092	*χ* ^2^_(1)_ = 16.11 P < **0.001**
4 September 2020	*χ* ^2^_(1)_ = 33.92 P < **0.001**	*χ* ^2^_(2)_ = 26.13 P < **0.001**	*χ* ^2^_(2)_ = 13.38 P = **0.001**	*χ* ^2^_(1)_ = 24.65 P < **0.001**
17 August 2021	*χ* ^2^_(1)_ = 36.44 P < **0.001**	*χ* ^2^_(2)_ = 21.48 P < **0.001**	*χ* ^2^_(2)_ = 29.54 P < **0.001**	*χ* ^2^_(1)_ = 28.45 P < **0.001**
30 August 2021	*χ* ^2^_(1)_ = 28.67 P < **0.001**	*χ* ^2^_(2)_ = 16.11 P < **0.001**	*χ* ^2^_(2)_ = 3.28 P = 0.194	*χ* ^2^_(1)_ = 24.21 P < **0.001**

Significant P values are boldfaced.

### Spectral reflectance

**Figure 2:**
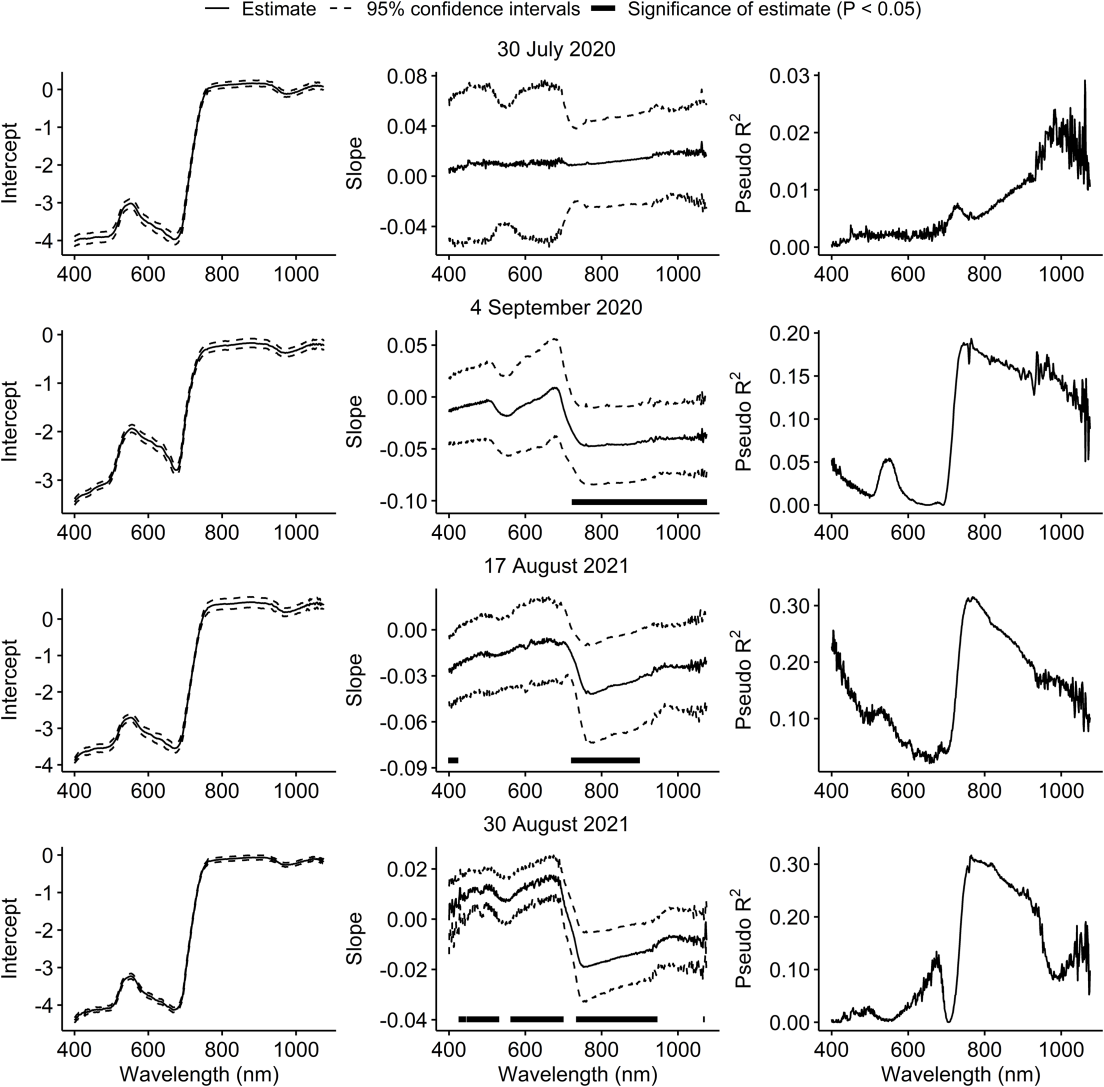
Estimates and 95% confidence intervals of intercepts and slopes, and pseudo coefficients of determination (R^2^) from beta regression models testing the effect of feeding injury from *Popillia japonica* on canopy spectral reflectance in plots of caged soybean plants in the field across 676 narrowband wavelengths on two sample dates in each of 2020 and 2021. Black horizontal bars within graphs in the middle column indicate that estimates of slopes for wavelengths differ from zero according to partial Wald tests (P < 0.05).

On 30 July 2020, spectral reflectance of the soybean canopy was not affected by injury from *P. japonica*. On 4 September 2020, a significant decrease in reflectance was observed at wavelengths above 723 nm. In 2021, a significant increase in reflectance was observed at wavelengths below 420 nm, and a significant decrease in reflectance from 722 to 898 nm on 17 August. On 30 August 2021, a significant increase in reflectance was generally observed from 427 to 529 nm and from 563 to 698 nm, and a significant decrease in reflectance from 735 to 944 nm (**Figure 2**). Across the three sample dates with significant effects of injury from *P. japonica* on spectral reflectance, the highest pseudo coefficients of determination (R^2^) were observed for wavelengths around the 780 nm region (**Figure 2**).

The vegetation indices were generally not affected by injury from *P. japonica* in 2020, except for MCARI on 4 September 2020 (**Table 3**). On this date, injury from *P. japonica* significantly decreased MCARI. In 2021, a significant reduction was also observed for NDVI, GNDVI (Green Normalized Difference Vegetation Index) and NDRE (Normalized Difference Red Edge) with increasing injury on 30 August (**Table 3**). Pseudo coefficients of determination (R^2^) values ranged from 0.001 to 0.172 in 2020, and from 0.003 to 0.304 in 2021 (**Table 3**). Overall, higher pseudo R^2^ were observed around 780 nm.

**Table 3:** Summary outputs of beta regression models testing the effects of *Popillia japonica* feeding injury on vegetation indices from canopy spectral reflectance in plots of caged soybean plants in the field during 2020 and 2021 in Saint Paul, MN and Rosemount, MN, respectively.

Date	Index	Parameter	Estimate	Std. Error	z value	P value	Pseudo R^2^
30 July 2020	NDVI	Intercept	2.58	0.06	41.33	**<0.001**	0.002
		Slope	-0.01	0.03	-0.26	0.792	
	NDRE	Intercept	1.11	0.05	23.19	**<0.001**	0.002
		Slope	-0.01	0.02	-0.37	0.713	
	GNDVI	Intercept	1.65	0.05	30.25	**<0.001**	0.001
		Slope	-0.01	0.03	-0.26	0.798	
	MCARI	Intercept	-2.66	0.06	-45.21	**<0.001**	0.003
		Slope	0.01	0.02	0.57	0.568	
4 September 2020	NDVI	Intercept	1.18	0.06	19.98	**<0.001**	0.076
		Slope	-0.04	0.03	-1.45	0.146	
	NDRE	Intercept	-0.48	0.06	-8.34	**<0.001**	0.037
		Slope	-0.03	0.02	-1.02	0.307	
	GNDVI	Intercept	0.23	0.04	5.55	**<0.001**	0.010
		Slope	-0.01	0.02	-0.47	0.639	
	MCARI	Intercept	-1.28	0.05	-24.07	**<0.001**	0.172
		Slope	-0.05	0.02	-2.60	**0.009**	
17 August 2021	NDVI	Intercept	2.28	0.09	26.72	**<0.001**	0.017
		Slope	-0.01	0.02	-0.72	0.473	
	NDRE	Intercept	0.95	0.07	12.79	**<0.001**	0.037
		Slope	-0.02	0.01	-1.25	0.210	
	GNDVI	Intercept	1.48	0.08	19.27	**<0.001**	0.003
		Slope	-0.01	0.01	-0.63	0.527	
	MCARI	Intercept	-2.48	0.06	-44.07	**<0.001**	0.047
		Slope	-0.01	0.01	-0.86	0.387	
30 August 2021	NDVI	Intercept	2.58	0.03	96.31	**<0.001**	0.304
		Slope	-0.02	0.00	-8.64	**<0.001**	
	NDRE	Intercept	1.12	0.03	37.46	**<0.001**	0.115
		Slope	-0.01	0.00	-3.82	**<0.001**	
	GNDVI	Intercept	1.74	0.03	60.07	**<0.001**	0.136
		Slope	-0.02	0.00	-5.81	**<0.001**	
	MCARI	Intercept	-2.82	0.05	-56.39	**<0.001**	0.079
		Slope	-0.01	0.01	-1.41	0.158	

Significant P values are boldfaced.

### Total yield and yield components

In 2020, injury from *P. japonica* adults did not affect seed number (mean: 2.52 x 10^7^; range: 1.86 x 10^5^ – 3.04 x 10^7^), 100-seed weight (mean: 16.60 g; range: 13.92 – 18.70 g), or total yield (mean: 4.17 ton per ha; range: 3.21 – 5.05 ton per ha) of soybean (**Table 4**). A similar result was found in 2021 for seed number (mean: 1.90 x 10^7^; range: 1.39 x 10^7^ – 2.28 x 10^7^) and total yield (mean: 3.24 ton per ha; range: 2.55 – 4.01 ton per ha). However, a significant effect was detected for 100-seed weight (mean: 17.06 g; range: 15.55 – 18.36 g), which decreased 1.6 g for every 10% increase in injury (**Table 4**).

**Table 4:** Summary outputs from general linear models estimating the effects of feeding injury (%) from *Popillia japonica* adults on seed number (seeds/ha x 10^6^), 100-seed weight (g) and total yield (ton/ha) in plots of caged soybean plants in the field during 2020 (n = 28) and 2021 (n = 17) in Saint Paul, MN and Rosemount, MN, respectively.

Factor	Parameter	Estimate	Std. Error	t value	P value
2020					
Seed number	Intercept	24.44	0.78	31.23	< **0.001**
	Slope	0.39	0.33	1.20	0.242
100-seed weight	Intercept	16.97	0.30	56.68	< **0.001**
	Slope	-0.21	0.13	-1.63	0.114
Total yield	Intercept	4.14	0.13	31.09	< **0.001**
	Slope	0.01	0.06	0.23	0.819
2021					
Seed number	Intercept	19.46	0.70	27.71	< **0.001**
	Slope	-0.16	0.16	-1.05	0.309
100-seed weight	Intercept	17.46	0.14	120.07	< **0.001**
	Slope	-0.14	0.03	-4.37	< **0.001**
Total yield	Intercept	3.39	0.12	29.25	< **0.001**
	Slope	-0.05	0.03	-2.09	0.054

Significant P values are boldfaced.

## Discussion

*Popillia japonica* is an invasive insect species of global concern for food crops in North America (1, 2) and, more recently, Europe (4). In this study, feeding injury by *P. japonica* was detectable and greater in the upper stratum of the canopy of soybean, with potential effects on canopy spectral reflectance and minimal effects on total yield and seed quality. This is consistent with the top-down feeding patterns well documented for *P. japonica* on a range of host plants (1).

Overall mean canopy injury ranged from 0.23 to 6.26%. The levels of injury attained in this study are representative of levels of *P. japonica* injury observed in soybean fields in the Midwest (41, 42). Such levels of injury (i.e., overall mean canopy injury < 15%) are also commonly observed in other crops fed on by *P. japonica* (28, 43). In soybean, typical infestation levels of *P. japonica* may not be a threat to yield (11). However, *P. japonica* is part of a complex of defoliating pests that, in combination, can result in defoliation greater than the economic thresholds currently adopted in the Midwest (i.e., 30% before bloom or 20% from bloom to pod fill (6)).

Leaf injury reduces photosynthetic area of plants and can cause disturbance of physiological processes including water and nutrient transportation, as well as eliciting the expression of defense responses, which are energetically costly and therefore reduce plant efficiency (9). *Popillia japonica* feeding was previously found to increase transpiration and consequently water loss in soybean leaflets without affecting carbon assimilation rates or photosynthetic efficiency (9). However, physiological effects observed at the leaf level are not necessarily reflected at the canopy level. For example, Ostlie and Pedigo (1984) (44) observed higher transpiration from soybean leaflets following artificial defoliation; but artificial defoliation or actual feeding by the green cloverworm, *Hypena scabra* (Fabricius) (Lepidoptera: Noctuidae), and the cabbage looper, *Trichoplusia ni* (Hubner) (Lepidoptera: Noctuidae), actually decreased canopy transpiration. Similarly, Klubertanz et al. (1996) (45) observed higher soil moisture in potted soybean plants following artificial defoliation.

The morphophysiological changes of plants caused by biotic or abiotic stresses can alter the spectral reflectance of plants including the visible and near infra-red ranges of the electromagnetic spectrum (15). For insect pests, this effect seems to be density-dependent as shown for *A. glycines* (20) and two lepidopteran pests (26) in soybean. In this study, a significant increase in soybean canopy reflectance of plants fed on by *P. japonica* at wavelengths in the visible range was observed, but this effect was inconsistent. However, a decrease in reflectance in the near infra-red associated with an increase in canopy injury from *P. japonica* was observed in three of the four dates across the two years. Lack of spectral response on the first sample date in 2020 was likely due to the low levels of injury observed on this date (**Figure 1**). Similarly, feeding injury by two lepidopteran species increased the visible and decreased near infra-red reflectance of soybean leaves in a greenhouse experiment (26). In contrast, an increase in both visible and near infra-red reflectance of leaves of peanut, *Arachis hypogaea* (L.) (Fabales: Fabaceae), was observed following injury by *Stegasta bosqueella* (Chambers) (Lepidoptera: Gelechiidae) and *S. cosmioides* (Walker) in the greenhouse (23).

Feeding injury from *P. japonica* to soybean tended to reduce spectral reflectance in the canopy in near infra-red wavelengths (~700 to 1000 nm). Furthermore, higher pseudo coefficients of determination (R^2^) around 780 nm indicate that this region is optimal for the detection of *P. japonica* injury in soybean. Canopy reflectance at 780 nm has also been shown to be optimal for the detection of *A. glycines* in soybean (46). Although *A. glycines* was present at densities lower than 20 aphids per plant in some plots used in this study, this effect was non-significant across all dates. For this reason, the results presented here are due to the effects of *P. japonica* injury alone. This corroborates previous findings indicating that *A. glycines* at densities lower than 20 aphids per plant result in negligible effects on soybean canopy reflectance (20, 32). Nevertheless, such overlapping effects on canopy spectral reflectance around 780 nm suggests that *P. japonica* feeding resulting in mean canopy injury of ≥ 5% may confound the detection of *A. glycines* or other herbivores in soybean. Thus, further investigation of spectral data that includes *P. japonica* injury coincident with other pests such as *A. glycines* is needed.

Inconsistent results were observed for the vegetation indices evaluated for the effects of *P. japonica* injury on soybean canopy reflectance. This lack of a consistent effect is probably because the vegetation indices tested here incorporate reflectance from the visible spectrum. Previous studies found the vegetation index NDVI is associated with the distribution of three lepidopteran pests, but not with their feeding injury in soybean fields (25). In cotton, differences in NDVI were observed for plants fed on by *Spodoptera exigua* (Hubner) (Lepidoptera: Noctuidae), but not for *T. ni* in the field (47).

Taken together, these findings indicate that plant spectral responses to defoliation are likely species-dependent, and care should be taken when generalizing across species of plants and defoliators. Furthermore, some of the results documented in the cited literature come from leaf measurements performed in the laboratory. Comparisons of canopy spectral reflectance obtained in the field to laboratory measurements can further create discrepancies between studies.

In this study, effects of *P. japonica* feeding injury on total yield were not observed, but a reduction in seed size was detected in one of the two years. Although soybean is tolerant to defoliation (48), the spatiotemporal distribution of canopy defoliation seems to have a differential impact on soybean yield. The intensity of feeding injury from *P. japonica* was quantified among strata of the soybean canopy more thoroughly than in previous studies. The lack of an effect of *P. japonica* feeding on total soybean yield confirms that contemporary soybean varieties likely respond similarly to this pest as those studied in the past.

In conclusion, near infra-red wavelengths may hold promise for remote sensing of *P. japonica* feeding injury in soybean. Because remote sensing can also be affected by other soybean pests, further studies incorporating near infra-red wavelengths and standard red, green and blue (RGB) imagery to differentiate *P. japonica* injury from that of other defoliators in soybean are needed. These results can facilitate refinement of management recommendations for *P. japonica* in soybean.

## Data availability statement

The raw data supporting the conclusions of this article will be made available by the authors, without undue reservation.

## Author contributions

AR, investigation, formal analysis, writing (original draft, review, and editing). TC, conceptualization, methodology, investigation, writing (review and editing). IM, conceptualization, writing (review and editing). RK, project administration, funding acquisition, conceptualization, methodology, writing (original draft, review, and editing). All authors contributed to the article and approved the submitted version.

## Funding

Funding for this research was provided by the Minnesota Soybean Research and Promotion Council and the Minnesota Invasive Terrestrial Plants and Pests Center through the Minnesota Environment and Natural Resources Trust Fund.

## Acknowledgments

We thank Rosa (Tina) Lozano, Rafael Carlesso Aita, James Menger, Grace Doyle, Samantha Janecek, Gunnar Morris, Ellen Adjeiwaa and Gloria Melotto for their help during data collection.

## Conflict of interest

The authors declare that the research was conducted in the absence of any commercial or financial relationships that could be construed as a potential conflict of interest.

## Publisher’s note

All claims expressed in this article are solely those of the authors and do not necessarily represent those of their affiliated organizations, or those of the publisher, the editors and the reviewers. Any product that may be evaluated in this article, or claim that may be made by its manufacturer, is not guaranteed or endorsed by the publisher.
